# Supplementary material for: Comparative genomics provides new insights into the diversity, physiology, and sexuality of the only industrially exploited tremellomycete: Phaffia rhodozyma
Source: BMC Genomics. 2016 Nov 9;17:901. doi: 10.1186/s12864-016-3244-7 (PMC5103461; doi:10.1186/s12864-016-3244-7)
Supplement: Additional file 6: — List of orphan genes with links to PFAM (related to Additional file 1: Table S1). (ZIP 1428 kb) [file 12864_2016_3244_MOESM6_ESM.zip › BLAST_HTML_FTR/G02875_P.html]

BLAST Search Results


```
BLASTP 2.2.27+


Reference:
Stephen F. Altschul, Thomas L. Madden, Alejandro A. Schäffer,
Jinghui Zhang, Zheng Zhang, Webb Miller, and David J. Lipman (1997),
"Gapped BLAST and PSI-BLAST: a new generation of protein database
search programs", Nucleic Acids Res. 25:3389-3402.


Reference for
composition-based statistics:
Alejandro A. Schäffer, L. Aravind, Thomas L. Madden, Sergei
Shavirin, John L. Spouge, Yuri I. Wolf, Eugene V. Koonin, and
Stephen F. Altschul (2001), "Improving the accuracy of PSI-BLAST
protein database searches with composition-based statistics and
other refinements", Nucleic Acids Res. 29:2994-3005.


Database: nr
           71,551,133 sequences; 26,053,659,533 total letters


Query= G02875_P

Length=477
                                                                      Score     E
Sequences producing significant alignments:                          (Bits)  Value

emb|CDZ98041.1|  hypothetical protein [Xanthophyllomyces dendrorh...   966    0.0  
ref|XP_003370224.1|  hypothetical protein Tsp_10833 [Trichinella ...  40.8    3.6  
ref|WP_042925535.1|  hypothetical protein [Rhodococcus qingshengi...  38.9    5.2  


 >emb|CDZ98041.1| hypothetical protein [Xanthophyllomyces dendrorhous]
Length=476

 Score =  966 bits (2497),  Expect = 0.0, Method: Compositional matrix adjust.
 Identities = 476/476 (100%), Positives = 476/476 (100%), Gaps = 0/476 (0%)

Query  1    MTSCAPPESTSSQKTVRFTTALAPMLHPAPLQTDRQAIRKYPVSVPFHSHNRRQATLQLP  60
            MTSCAPPESTSSQKTVRFTTALAPMLHPAPLQTDRQAIRKYPVSVPFHSHNRRQATLQLP
Sbjct  1    MTSCAPPESTSSQKTVRFTTALAPMLHPAPLQTDRQAIRKYPVSVPFHSHNRRQATLQLP  60

Query  61   VRVSHVPYASLPAMTSAGGAHADHQSEGEGEVEVEGAGVEVTRSKTSRRRRVSGGRRIGG  120
            VRVSHVPYASLPAMTSAGGAHADHQSEGEGEVEVEGAGVEVTRSKTSRRRRVSGGRRIGG
Sbjct  61   VRVSHVPYASLPAMTSAGGAHADHQSEGEGEVEVEGAGVEVTRSKTSRRRRVSGGRRIGG  120

Query  121  PRANIKYSGTGTTEGSLGRRNALKLLLDARKENDGSKSSNKPLFTVDSLSPLQSLVASLS  180
            PRANIKYSGTGTTEGSLGRRNALKLLLDARKENDGSKSSNKPLFTVDSLSPLQSLVASLS
Sbjct  121  PRANIKYSGTGTTEGSLGRRNALKLLLDARKENDGSKSSNKPLFTVDSLSPLQSLVASLS  180

Query  181  SYPSSYGLRCFSAFADSPLSSCTSSPLLSSGSVNILEANPWIRPLTSAILAQPVPIPLHG  240
            SYPSSYGLRCFSAFADSPLSSCTSSPLLSSGSVNILEANPWIRPLTSAILAQPVPIPLHG
Sbjct  181  SYPSSYGLRCFSAFADSPLSSCTSSPLLSSGSVNILEANPWIRPLTSAILAQPVPIPLHG  240

Query  241  HQLSRSPLLDSTCTFSEFRFHSESPRLDTAEAGSGSSHLKRMDSHVFRALNAFDQTDASE  300
            HQLSRSPLLDSTCTFSEFRFHSESPRLDTAEAGSGSSHLKRMDSHVFRALNAFDQTDASE
Sbjct  241  HQLSRSPLLDSTCTFSEFRFHSESPRLDTAEAGSGSSHLKRMDSHVFRALNAFDQTDASE  300

Query  301  PAGNVGLVSPWIDDQRGDTKGLLKKRSIWTRSLSENGSISESSVHTSDTSTTDSVTSDCD  360
            PAGNVGLVSPWIDDQRGDTKGLLKKRSIWTRSLSENGSISESSVHTSDTSTTDSVTSDCD
Sbjct  301  PAGNVGLVSPWIDDQRGDTKGLLKKRSIWTRSLSENGSISESSVHTSDTSTTDSVTSDCD  360

Query  361  SASTAATSEAEFDPVGLGFYNAPNNFNSRPYAGLKPILLCRTSARLAPLPYPTQPLDGQN  420
            SASTAATSEAEFDPVGLGFYNAPNNFNSRPYAGLKPILLCRTSARLAPLPYPTQPLDGQN
Sbjct  361  SASTAATSEAEFDPVGLGFYNAPNNFNSRPYAGLKPILLCRTSARLAPLPYPTQPLDGQN  420

Query  421  KVRKIHHPIPVRPFVPSDSPLILTDILDEMLSSWPTPEFSMTSAFSGPSEEGVQLD  476
            KVRKIHHPIPVRPFVPSDSPLILTDILDEMLSSWPTPEFSMTSAFSGPSEEGVQLD
Sbjct  421  KVRKIHHPIPVRPFVPSDSPLILTDILDEMLSSWPTPEFSMTSAFSGPSEEGVQLD  476


>ref|XP_003370224.1| hypothetical protein Tsp_10833 [Trichinella spiralis]
 gb|EFV50312.1| hypothetical protein Tsp_10833 [Trichinella spiralis]
Length=922

 Score = 40.8 bits (94),  Expect = 3.6, Method: Compositional matrix adjust.
 Identities = 27/77 (35%), Positives = 43/77 (56%), Gaps = 5/77 (6%)

Query  99   VEVTRSKTSRRR--RVSGGRRIGGPRANIKYSGTGTTEGSLGRRNALKLLLDARKENDGS  156
            +E  +++   RR   VSG       +ANI +S    T  SLG+R+ALKL  +A +E   +
Sbjct  122  IEFHQNQEESRRILSVSGKTLCDFLKANIPFSCNVKTSTSLGKRSALKLNAEANQEGANA  181

Query  157  KSSNKPLFTVDSLSPLQ  173
            K+ N P    +S+SP++
Sbjct  182  KTGNSPQ---ESMSPIE  195


>ref|WP_042925535.1| hypothetical protein [Rhodococcus qingshengii]
 gb|KDQ01198.1| hypothetical protein EN35_24845 [Rhodococcus qingshengii]
Length=172

 Score = 38.9 bits (89),  Expect = 5.2, Method: Compositional matrix adjust.
 Identities = 30/92 (33%), Positives = 40/92 (43%), Gaps = 7/92 (8%)

Query  57   LQLPVRVSHVPY-ASLPAMTSAGGAHADHQSEGEGEVEVEGAGVEVTRSKTSRRRRVSGG  115
            L L + +SH+P+  + P  + A GAH  H S G    E+       + SKT      +  
Sbjct  63   LNLSIILSHIPFEKAFPKESDAAGAHKIHLSVGSHHKELR------SPSKTPPTEAAAWA  116

Query  116  RRIGGPRANIKYSGTGTTEGSLGRRNALKLLL  147
              I GP AN  YS       S+GR  AL   L
Sbjct  117  AAIFGPWANDAYSLGAIVRNSIGRPPALHFAL  148


Lambda      K        H        a         alpha
   0.314    0.129    0.379    0.792     4.96 

Gapped
Lambda      K        H        a         alpha    sigma
   0.267   0.0410    0.140     1.90     42.6     43.6 

Effective search space used: 4818161321596


  Database: nr
    Posted date:  Sep 23, 2015 12:05 AM
  Number of letters in database: 26,053,659,533
  Number of sequences in database:  71,551,133


Matrix: BLOSUM62
Gap Penalties: Existence: 11, Extension: 1
Neighboring words threshold: 11
Window for multiple hits: 40
```
